# Female susceptibility to cancer and other diseases as indicated by British and European mortality rates.

**DOI:** 10.1038/bjc.1969.35

**Published:** 1969-06

**Authors:** P. Stocks


### FEMALE SUSCEPTIBILITY TO CANCER AND OTHER DISEASES AS INDICATED BY BRITISH AND EUROPEAN MORTALITY RATES

P. STOCKS*

Received for publication February 3, 1969

FOR most of the causes of death which affect both sexes males register higher rates than females, and for only about 5 per cent of the total deaths of males at ages 55–74 in England and Wales does the corresponding female death rate exceed that of the males.

An example of apparent female susceptibility which was noticed when statistics of deaths by sex, age and cause began to be analysed was seen in whooping cough. In 1936–39 when deaths from that disease were averaging about 1500 annually the female/male sex ratio for death rates in the first year of life was about 1·2, increasing to 1·5 and over as age advanced. Amongst possible reasons suggested for the female excess were higher risk of a fatal issue amongst female children contracting the disease and a greater danger to females of catching the disease from children who had to be looked after by them. Since then preventive measures including inoculation have reduced the annual deaths to about 20, but since notifications of whooping cough are being recorded by sex and age, about 2500 annually, it is possible to ascertain the sex differential in rates of incidence. A female excess is found to be present from the first years of life and to increase from 5 per cent to over 100 per cent in adults, just as was the case for death rates in pre-immunisation years. This is shown in Table I for the period 1957–66.

TABLE I.—*Sex-ratio of Whooping Cough Notifications in England and Wales in* 1957–66 *at Different Ages*

| | Female/male ratios at ages: | | | | | |
|---|---|---|---|---|---|---|
| | 0– | 1– | 5– | 10– | 15– | 25 and over |
| Whooping cough (056) . | 1·05 | 1·13 | 1·21 | 1·22 | 1·75 | 2·08 |

The trend of the rates suggests differential exposure of the sexes to cross-infection as the reason rather than a genetic factor located in the X-chromosomes which would be expected to operate fairly uniformly from the start of life, but this is conjectural.

This example of whooping cough poses similar problems for other diseases where females suffer higher rates of dying than males at certain ages, and the present study examines the age patterns of the sex ratios for such diseases shown by the mortality statistics of England and Wales and other countries where the information is available.

Table II lists the diseases affecting organs not peculiar to females which have female/male sex-ratios of their death rates in England and Wales in excess of

*Address: 34 Brompton Avenue, Colwyn Bay, North Wales.

unity at some or all ages after 35. The ratios are between the mean annual death rates in the period 1963–66, that is the total deaths divided by the total population at risk, for each sex. For four of the 18 groups a longer period of years has been used to eliminate effects of chance year-to-year variations in annual deaths when the numbers of these are small. For convenience the last column shows an Index defined as 100 times the average of the sex-ratios at ages 45–54, 55–64 and 65–74. The magnitude of the indices ranges from 337 to 100 and the changes in the sex-ratio according to age are of several kinds, and the 18 diseases have been grouped accordingly for the sake of clarity.

In a developed society in times of peace differences in risks of death from specific diseases to which the two sexes are exposed may be of importance in some occupational groups but they contribute little to the overall differences in sex-ratios in the population as a whole. The dangers of childbearing have become inconsiderable as causes of death, but for certain cancers and disorders of the endocrine and biliary system, diseases of the blood-forming organs, rheumatoid arthritis and some circulatory and nervous conditions females appear to be more vulnerable than males. In some cases this is presumably due to peculiarities in the control exercised by genes in the X-chromosomes of which an extra one occurs in females. For example, more demands are made upon the thyroid, pituitary and haemic system by the female organism, and any enzyme control from genes in the extra chromosome peculiar to women may differ in some way from that exercised by genes in the other chromosomes which occur in the cells of both sexes. It is hardly surprising that at least five of the diseases in Table II concern the endocrine glands, three are affections of the blood and blood vessels and three of the liver and biliary systems.

Four groups of malignant neoplasms appear in the list and their association with non-malignant conditions of the same organs may furnish some clues to the genetic factors responsible for the high female susceptibility to those particular cancers. These are discussed in detail below but the table shows that the indices for the cancer groups range from 182 to 104, and that in behaviour of the sex-ratio according to age they have nothing in common. For thyroid cancer female excess is high throughout life, for the biliary passages it appears only after 45, for intestine it disappears after 60 and for malignant melanoma of the skin it diminishes with advancing age.

*Diseases showing excess of female over male death rates*

*Thyroid diseases other than cancer* (international numbers 250–254) show a higher level of female excess in rates of dying than any other group, with ratios around 4 except for a temporary fall between ages 45 and 60 which may be connected with the menopause. This high susceptibility of women to disorders resulting in death derives presumably from genes in the additional X-chromosome since the gland has some important functions connected with childbearing and peculiar to females.

*Cancer of the thyroid* (194) manifests a similar female excess at each age with sex-ratios around 2 but with a depression at ages 45–60 as for the other thyroid diseases. The index, 182, compares with 337 in that group. Since the cancer death rates are not very large a longer period of 14 years (1950–63) has been used, the data for England and Wales being extracted from the international compilations by Segi and Kurihara (1966). At ages 25–34 the sex-ratio was 2·02, and at

TABLE II.—*Diseases with Female Susceptibility Indicated by Death Rates in England and Wales in* 1963–66

| Disease group | International No. | Ratio, female/male death rate at the specified age-group 35– | 45– | 55– | 65– | 75+ | Index 100 × average ratio at 45–74 |
|---|---|---|---|---|---|---|---|
| | | High female/male ratios at most ages | | | | | |
| Thyroid diseases (except cancer) | 250–4 | 4·42 | 2·83 | 3·84 | 4·93 | 4·35 | 337 |
| Cancer of thyroid* | 194 | 2·42 | 1·62 | 1·93 | 2·03 | 2·53 | 182 |
| Rheumatoid arthritis | 722 | 3·60 | 1·86 | 2·06 | 2·21 | 2·78 | 208 |
| Chronic rheumatic heart | 410–6 | 1·45 | 1·56 | 1·57 | 1·44 | 1·51 | 152 |
| Obesity | 287 | 2·51 | 2·19 | 2·00 | 2·53 | 2·46 | 224 |
| Diseases of gall bladder and ducts (except cancer) | 584–6 | 1·45 | 1·47 | 1·46 | 1·19 | 1·12 | 137 |
| Subarachnoid haemorrhage | 330 | 1·12 | 1·30 | 1·24 | 1·48 | 1·67 | 134 |
| Anaemias, purpura, diseases of spleen etc. | 290–3, 296, 298–9 | 1·65 | 1·45 | 1·17 | 1·18 | 1·16 | 127 |
| | | High female/male ratios until age 60 | | | | | |
| Pituitary and adrenal diseases† | 272, 274 | 1·59 | 1·53 | 1·15 | 0·86 | 0·67 | 118 |
| Asthma | 241 | 1·54 | 1·31 | 1·10 | 0·87 | 0·96 | 109 |
| Cancer of intestines except rectum* | 152–3 | 1·20 | 1·20 | 1·05 | 0·87 | 0·99 | 104 |
| Multiple sclerosis | 345 | 1·61 | 1·56 | 1·31 | 0·96 | 0·82 | 127 |
| | | High female/male ratios after 55 or 65 | | | | | |
| Cancer of biliary passages | 155·1–155·8 | 0·59 | 1·07 | 1·36 | 1·29 | 1·30 | 124 |
| Diabetes mellitus | 260 | 0·55 | 0·74 | 1·19 | 1·42 | 1·14 | 112 |
| | | Moderate female excess at most ages | | | | | |
| Malignant melanoma of skin | 190 | 1·27 | 1·11 | 1·09 | 1·08 | 1·00 | 109 |
| Chronic endocarditis, not rheumatic and not aortic valves† | 421·0, 421·2, 421·3, 4 | 1·04 | 1·15 | 0·99 | 1·07 | 1·28 | 107 |
| Liver diseases except cirrhosis and cancer | 580, 582–3 | 1·04 | 1·26 | 0·99 | 1·03 | 0·81 | 109 |
| Diseases of veins, embolism | 460–6 | 1·57 | 1·14 | 0·85 | 1·00 | 1·08 | 100 |

* Averages for 10 years 1957–66.
† Averages for 14 years 1950–63.

successive 5-year age groups from 35–39 onwards the ratios were 2·6, 2·5, 1·5, 1·7, 1·7, 2·2, 1·9, 2·1, 2·1, 2·3, 3·4, revealing more clearly the drop in ratio after 45 to 1·5 and 1·7 and a return to the average 2·2 level after age 60. In Fig. 1 the death rates for each sex in England and Wales are depicted on a logarithmic scale so that the vertical gaps between the two curves conveniently represent the actual female/male ratios at each age. The gap is seen to narrow after age 40 and widen again later without obvious disturbance of the male mortality curve, indicating a temporary lessening of female susceptibility between 40 and 60.

In Table III the mean annual death rates of women from thyroid cancer per 10 million at risk in 1954–61 are shown for 13 countries of Europe and the ratios of these rates to those of men are compared at different ages. The countries are ranked in descending order of the index in the last column, defined as before as 100 times the mean of the 3 ratios between 45 and 74. The indices range from 313 in Ireland to 130 in Austria, and it is remarkable that the first seven countries are all in northern Europe whereas five of the six countries with indices below 175 are countries in mid-Europe, the only one out of place being Norway (142). There seems to be a climatic factor involved with strong effect upon female vulnerability to the disease. The higher ratios in the north are not merely due to higher female death rates with the male rates unaffected; there are in fact two countries, Switzerland and Austria, with much the highest female rates at every age up to 75 but also the lowest female/male indices, these countries being mountainous with high prevalence of simple goitre. The averages of the rates for each sex in four parts of Europe are shown in Table IV. The Swiss and Austrian rates far exceed those of the other areas for each sex. For males the Scandinavian rates exceed those of Britain at all ages and the mid-Europe rates at all ages except 55–64, but the British rates show no excess over mid-Europe. For females both Scandinavian and British rates exceed the mid-European at ages over 45 and a strong northern excess is evident after 65.

That the female excess in death rates is not to be explained by sex differences in fatality is shown by the incidence rates derived from cancer registration in Denmark during 1943–57 (Clemmesen, 1964) which yield the following female/male sex-ratios at successive age groups from 35–44: 1·6, 1·9, 5·0, 2·4, 2·9. These would lead to an index of 310 compared with 200 for death rates in that country.

TABLE III.—*Death Rates of Women from Cancer of the Thyroid in* 1954–61 *and Sex-ratios in* 13 *Countries*

| Countries | Mean annual female rates per 10 million | | | | | Ratios to male rates at specified ages | | | | | Index at 45–74 |
|---|---|---|---|---|---|---|---|---|---|---|---|
| | 35– | 45– | 55– | 65– | 75+ | 35– | 45– | 55– | 65– | 75+ | |
| Eire and N. Ireland | 4 | 15 | 34 | 80 | 84 | 1·8 | 2·7 | 3·3 | 3·4 | 3·2 | 313 |
| Sweden | 4 | 8 | 21 | 89 | 136 | 1·1 | 1·9 | 1·7 | 3·0 | 3·7 | 220 |
| Scotland | 2 | 11 | 27 | 69 | 89 | 1·5 | 0·9 | 2·4 | 2·9 | 1·6 | 207 |
| Denmark | 2 | 12 | 34 | 56 | 140 | 1·7 | 2·2 | 2·4 | 1·4 | 2·4 | 200 |
| Finland | 5 | 12 | 30 | 51 | 96 | 1·0 | 1·3 | 1·8 | 2·7 | 1·6 | 193 |
| England and Wales | 2 | 8 | 21 | 45 | 74 | 2·0 | 1·8 | 1·9 | 1·9 | 3·6 | 189 |
| Netherlands and Belgium | 3 | 9 | 21 | 47 | 62 | 2·1 | 1·6 | 1·8 | 2·0 | 2·9 | 177 |
| France | 2 | 6 | 20 | 38 | 48 | 1·7 | 1·4 | 1·4 | 2·3 | 2·2 | 168 |
| Italy | 3 | 9 | 23 | 47 | 55 | 1·4 | 1·3 | 1·5 | 2·1 | 1·8 | 161 |
| Germany, F.R. | 3 | 12 | 29 | 62 | 82 | 1·2 | 1·5 | 1·2 | 1·6 | 2·1 | 142 |
| Norway* | 2 | 7 | 25 | 66 | 188 | 1·9 | 1·1 | 1·6 | 1·6 | 2·7 | 142 |
| Switzerland | 5 | 36 | 62 | 126 | 149 | 3·4 | 2·0 | 1·0 | 1·1 | 1·5 | 138 |
| Austria | 8 | 21 | 48 | 125 | 199 | 1·6 | 1·3 | 1·1 | 1·6 | 1·8 | 130 |

* Based on 12 years 1952–63

TABLE IV.—*Thyroid Cancer Death Rates in Parts of Europe, Mean Annual per* 10 *million in* 1954–61 (*Averages for Countries in Group*)

| | 35– | 45– | 55– | 65– | 75+ | Index 45–74 |
|---|---|---|---|---|---|---|
| | Males | | | | | |
| Switzerland and Austria | 3·1 | 17·6 | 54 | 95 | 126 | |
| Other mid-Europe | 1·8 | 6·2 | 17 | 26 | 48 | |
| British Isles | 1·8 | 6·3 | 11 | 24 | 31 | |
| Scandinavian countries | 2·6 | 7·0 | 15 | 36 | 49 | |
| | Females | | | | | |
| Switzerland and Austria | 6·3 | 28·8 | 50 | 125 | 174 | 130 |
| Other mid-Europe | 2·8 | 9·0 | 23 | 48 | 62 | 158 |
| British Isles | 2·5 | 11·5 | 29 | 69 | 82 | 244 |
| Scandinavian countries | 3·3 | 10·0 | 28 | 74 | 124 | 170 |

*Rheumatoid arthritis* (722) shows evidence of high female susceptibility with sex-ratios of 3·6 at 35–44, around 2 between 45 and 74 and 2·8 at ages 75 and over (index 218). The causes of this disease are still obscure and the reputed roles of heredity, infection, auto-immunity and failure of adaptation to stress are all in doubt. According to Copeman (1964) " a number of factors may act as predisposing or precipitating agents in the presence of a soil rendered vulnerable by genetic, environmental or metabolic influences ". There are, however, no external factors known which could account for the large sex difference in rates of dying from the disease. Lawrence (1961) calculated that the minimal prevalence of the disease in Great Britain was 2·1 per cent in men and 5·2 per cent in women, giving a sex-ratio similar to that of mortality found above. A genetic factor in the extra X-chromosome would seem likely to account for the high female susceptibility. It may be noted that osteo-arthritis, osteitis deformans and chronic diseases of the bones (723, 731, 733) show no such female excess at ages before 75 (index 98).

*Chronic rheumatic heart* (410–416) and other chronic endocarditis not involving the aortic valves (421·0, 421·2, 421·3, 421·4). The first of these groups, comprising deaths from chronic heart disease attributed by the certifier to rheumatic fever and from lesions of the mitral valve with no cause stated on the certificate, shows in Table II high female/male sex-ratios around 1·5 at every age (index 152). This contrasts with acute rheumatism (400–402) for which there is a male excess at every age under 70, and it is in even stronger contrast from chronic rheumatic disease involving the aortic valve (index 39). The various subdivisions of deaths classified as chronic rheumatic heart disease are distinguished in Table V where sex-ratios in the ten years 1957–66 have been calculated, and in the same table are shown acute rheumatism including chorea and the groups of other endocarditis with acute myocarditis and pericarditis.

Mitral disease has the highest index of female susceptibility (180) with ratios over 1·7 at each age under 75, but the small group with mitral involvement said to be of non-rheumatic origin shows little sex difference at those ages. Aortic valve disease affects males much more frequently than females, with indices of 39 for the deaths attributed to rheumatism as cause and 37 for the larger group of those not so attributed. The three groups of other chronic heart disease said to be of rheumatic origin all show considerable female excess with indices of 131 for endocarditis of the tricuspid or unspecified valve, 121 for myocarditis or pericarditis said to be rheumatic and 156 for the residual group of chronic rheumatic heart not precisely described. The corresponding small groups of endocarditis said to be of other than rheumatic cause and without mention of the mitral or aortic valve show female excess at 45–54 and after 75 but no appreciable sex difference at other ages (index 107).

Since active acute rheumatic fever produces higher death rates of males than of females at every age (index 70), it is evident from the table that women are peculiarly susceptible to chronic rheumatic affection of the mitral valve and men to any affection of the aortic valve, the indices for these valvular involvements differing very greatly in a proportion of about 5 to 1. Furthermore, since myocardial and other forms of chronic rheumatic heart affection not involving the aortic valve also show female excess with indices ranging from 122 to 156, the mitral valve is not the only part of the heart which is more vulnerable to disease among females.

TABLE V.—*Sex-ratios Between Mean Death Rates in* 1957–60 *in England and Wales for Chronic Rheumatic Heart Disease, Other Endocarditis and Acute Rheumatism*

| Categories of heart disease | International No. | No. of deaths at 35+ | Female/male sex-ratios 35– | 45– | 55– | 65– | 75+ | Index 45–74 |
|---|---|---|---|---|---|---|---|---|
| *Mitral valve* involved | | | | | | | | |
| Rheumatic or unstated | 410 | 45728 | 1·73 | 1·87 | 1·80 | 1·72 | 1·60 | 180 |
| Non-rheumatic cause | 421·0 | 2970 | — | 0·54 | 0·83 | 1·13 | 1·47 | 83 |
| *Aortic valve,* not mitral | | | | | | | | |
| Rheumatic cause | 411 | 4994 | 0·32 | 0·39 | 0·39 | 0·41 | 0·71 | 39 |
| Other or unspecified | 421·1 | 22399 | 0·24 | 0·27 | 0·29 | 0·54 | 0·80 | 37 |
| *Other chronic rheumatic* | | | | | | | | |
| Endocarditis | 412–4 | 3782 | 1·37 | 1·43 | 1·28 | 1·22 | 1·44 | 131 |
| Myocarditis, pericarditis | 415 | 1215 | 0·96 | 1·22 | 1·26 | 1·18 | 1·61 | 122 |
| Heart, unspecified | 416 | 11398 | 1·23 | 1·50 | 1·60 | 1·58 | 1·59 | 156 |
| *Non-rheumatic cause specified* | | | | | | | | |
| Endocarditis, except mitral or aortic | 421·2–421·4 | 5286 | 1·02 | 1·23 | 0·98 | 1·01 | 1·16 | 107 |
| *Acute rheumatism, active* | 400–2 | 730 | 0·89 | 0·92 | 0·57 | 0·60 | 0·73 | 70 |

*Multiple sclerosis* (345), from which there were 3207 deaths in England and Wales in 1963–66 showed high female susceptibility at ages up to 65 with sex-ratios of 1·61 at 35–44, 1·56 at 45–54 and 1·31 at 55–64, but no female excess after that (index 127). By way of contrast paralysis agitans (350) has a male excess at every age (index 73).

*Subarachnoid haemorrhage* (330) is shown in Table II to have a female excess in the death rates during 1963–66 which increases with advancing age, the successive sex-ratios being 1·22 at 35–44, 1·27 at 45–64, 1·48 at 65–74 and 1·67 after 75 (index 134). This differs remarkably from other cerebral haemorrhage, thrombosis, embolism and ill-defined vascular lesions of the central nervous system as can be seen in Table VI.

TABLE VI.—*Sex-ratios Between Mean Death Rates in* 1963–66 *in England and Wales for Vascular Lesions Affecting the Central Nervous System*

| Category of disease | International No. | No. of deaths at 35+ | Female/male sex-ratios 35– | 45– | 55– | 65– | 75+ | Index at 45–74 |
|---|---|---|---|---|---|---|---|---|
| Subarachnoid haemorrage | 330 | 14047 | 1·12 | 1·30 | 1·24 | 1·48 | 1·67 | 134 |
| Cerebral haemorrhage | 331 | 120310 | 0·77 | 0·99 | 0·81 | 0·86 | 1·02 | 89 |
| Cerebral thrombosis and embolism | 332 | 145300 | 1·03 | 0·67 | 0·62 | 0·72 | 0·94 | 67 |
| Other ill-defined vascular lesions | 333–4 | 25604 | 0·70 | 0·62 | 0·54 | 0·65 | 0·86 | 60 |

The aggregate numbers of deaths at age over 35 were greater for women than for men in each group, but that was due to the much larger female populations at risk at advanced ages and is no guide to the relative death rates of the two sexes. There were for example more deaths of women than of men from cerebral haemorrhage at each age group after 45.

The index for subarachnoid haemorrhage at ages 45–74 was 134 compared with 89, 67 and 60 for the other groups of cerebral lesions, and the high female susceptibility to the subarachnoid variety can hardly arise from an external cause and must be due to a tissue weakness peculiar to the female brain.

The geographical distribution of mortality from all forms of vascular lesions of the central nervous system combined has been examined in another paper (Stocks, 1968), but data were not available to allow distinction of subarachnoid haemorrhage from other varieties.

*Obesity* (287) has a remarkably uniform sex-ratio exceeding 2 at each age and an index of 224. The existence of such a steady level of female susceptibility throughout life could hardly arise from extrinsic factors such as dietary peculiarities of women's habits for the differential effects on mortality of the sexes would surely change with advancing age. It seems reasonable to attribute the cause to a controlling influence over the endocrine glands emanating from the extra X-chromosome.

*Pituitary and adrenal diseases* (272, 274) were responsible in the ten years 1957–66 for mean annual death rates as depicted in Fig. 1 at ages 25 onwards, based on 1688 deaths. Between 35 and 62 there was a pronounced excess of the female rate but at the two ends of life males had the higher rates. The female/male sex-ratio at ages 0–14 was 0·74, at 15–24 0·86 and at 25–34 0·82, but as seen in Table II the ratios at the next three age groups were 1·59, 1·53, 1·15, falling again below unity after 65. The high susceptibility of women to diseases of these glands between 35 and 55 arises presumably from the unusual demands on their activity by women during that period of life, and it contrasts with the depressed female/male ratio at 45–54 which has been noted for thyroid diseases.

Cancers of the pituitary and adrenals show no female excess in death rates at 45–74, the index in 1958–66 being 48 compared with 118 for other diseases of the glands.

*Cancer of the biliary passages* (155·1–155·8) is one of the very few sites of malignant neoplasms which shows evidence of female susceptibility, excluding of course the breast and organs peculiar to women. Table II shows that the female/male ratio between death rates in 1963–66 increased after age 40 to about 1·3 and then remained about that level (index 124).

Fig. 1 depicts the rates for each sex on a logarithmic scale. A similar pattern appears for non-malignant diseases of the gall bladder and ducts which are dealt with below.

The sex-ratios for primary cancer of the liver (155·0) are entirely different from those for the biliary passages. In England and Wales the liver group shows pronounced male excess with female/male ratios at age groups 35–44 onwards 0·43, 0·42, 0·40, 0·45, 0·56 compared with 0·59, 1·07, 1·36, 1·29, 1·30 for the duct cancers. For the two categories combined the ratios are close to unity in Great Britain but not in Ireland as a whole where the death rates in 1954–61 showed female excess at each age with indices about 125 as may be seen in Table VII.

No reason for the female susceptibility in the two parts of Ireland but not in Great Britain can be suggested, but the Netherlands has a very high index of 147 and the German Federal Republic 130. The Scandinavian countries, France, Switzerland and Italy show, like Great Britain, no female excess, but Austrian ratios show a U-shaped trend with female excess at the two ends of life (index 106).

Separation of primary liver cancer from malignant neoplasms of the gall bladder and bile ducts, not possible from the available data of the countries in Table VII, has been made below for death rates in cities of Latin America and for

FIG. 1.—Death rates for each sex in England and Wales from cancer of thyroid 1950–63, and bile ducts 1963–66, other diseases of pituitary and adrenals 1957–66.

(*a*) Cancer of thyroid.
(*b*) Cancer of gall bladder and bile ducts.
(*c*) Diseases of pituitary and adrenal (non-malignant).

*Vertical heights at different ages for graphs*

| | | 25– | 30– | 35– | 40– | 45– | 50– | 55– | 60– | 65– | 70– | 75– | 80– | 85– |
|---|---|---|---|---|---|---|---|---|---|---|---|---|---|---|
| *Cancer of thyroid* 1950–63 (per 10 million) | M | 2·2 | 2·2 | 5·2 | 11·0 | 25·9 | 45·2 | 70·0 | 88·6 | 142 | 173 | 214 | 225 | 166 |
| | F | 3·3 | 5·6 | 13·5 | 25·9 | 38·7 | 76·8 | 117 | 195 | 267 | 372 | 446 | 516 | 513 |
| *Cancer of bile ducts* 1963–66 (per million) | M | | | 4·14 | | 10·9 | | 34·1 | | 85·5 | | 157·3 | | |
| | F | | | 2·45 | | 11·7 | | 46·4 | | 110·0 | | 204·2 | | |
| *Diseases of pituitary and adrenal* 1957–66 (per million) | M | 1·1 | 2·0 | 1·6 | 2·3 | 2·8 | 4·45 | 6·34 | 10·3 | 13·1 | 15·8 | 15·3 | 12·6 | |
| | F | 1·2 | 1·4 | 2·4 | 3·9 | 4·4 | 6·63 | 8·13 | 10·6 | 11·4 | 13·6 | 9·0 | 9·9 | |

TABLE VII.—*Sex-ratios of Death Rates in* 1954–66 *for Cancers of the Liver and Biliary Passages Combined in Parts of Europe*

| Country | Female/male ratios at ages 35– | 45– | 55– | 65– | 75+ | Index 45–74 |
|---|---|---|---|---|---|---|
| England and Wales | 0·92 | 0·88 | 0·83 | 0·93 | 1·05 | 88 |
| Scotland | 0·96 | 0·94 | 0·87 | 1·00 | 1·01 | 94 |
| Northern Ireland | 1·66 | 1·29 | 1·33 | 1·12 | 1·27 | 125 |
| Eire (Irish Republic) | 0·99 | 1·21 | 1·19 | 1·31 | 0·88 | 124 |
| Scandinavia | 0·95 | 0·84 | 0·91 | 1·01 | 1·03 | 92 |
| Netherlands | 0·78 | 1·34 | 1·47 | 1·61 | 1·62 | 149 |
| Germany, F.R. | 1·00 | 1·32 | 1·28 | 1·32 | 1·47 | 130 |
| Austria | 1·49 | 1·09 | 0·95 | 1·14 | 1·29 | 106 |
| France | 1·01 | 0·78 | 0·72 | 0·79 | 0·98 | 76 |
| Switzerland | 1·33 | 1·03 | 0·95 | 0·65 | 1·06 | 88 |
| Italy | 0·90 | 0·84 | 0·84 | 0·95 | 1·09 | 88 |

morbidity rates extracted from cancer registration records in Denmark, Norway, Israel and Connecticut, the ratios expressing female in terms of male rates.

In 1962–64 clinical and post-morten investigations were made of the persons aged 15–74 who died in 10 Latin American cities and in Bristol and San Francisco, 169 of the deaths being classified as due to primary cancer of the liver and 295 as cancer of the biliary passages (Puffer and Wynne Griffith, 1967). The sex-ratios between the age-adjusted rates (with numbers of deaths in parentheses) were as follows: Primary liver 0·55; total biliary passages 1·96; gall bladder 4·50 (134); bile ducts 1·14 (58); ampulla of Vater 0·67 (31); site not determined 2·0 (72).

From the cancer register in Copenhagen average sex-ratios for Denmark in the 15 years 1943–57 (Clemmesen, 1964) were for the four age groups 35–44, 45–54, 55–64, 65–74; Primary liver 1·03; Biliary passages 2·37. From Connecticut in 1947–51 (Griswold *et al.*, 1955) the corresponding ratios were 0·65 and 1·82. Cancer registration in Norway (Norwegian Cancer Society, 1964) showed for the whole country in 1959–61 average sex-ratios at 45–54, 55–64 and 65–74 of 0·64 and 1·48 for the two cancer groups. In Israel in 1960–64 (Steinitz, 1967) the standardised rates at ages over 15 in the total Jewish population gave sex-ratios of 0·54 and 3·10 for those groups.

It is evident from the above that the female vulnerability to cancer of the biliary passages which was indicated by mortality in England and Wales is not accounted for by fatality differences but is even more pronounced in incidence rates, and the figures confirm that primary cancer of the liver is much more frequent in occurrence among males.

Cancer of the pancreas (157) shows no female excess in England and Wales, the ratios in 1963–66 at the 5 age groups being 0·58, 0·56, 0·56, 0·58, 0·69 (index 56).

*Diseases of the gall bladder and bile ducts* (584–586), *and of the liver* (*except cancer and cirrhosis*) (580, 582, 583).—Liver diseases except cirrhosis and cancer, consisting mainly of acute forms of hepatitis not included among the infective diseases, show a female excess (ratio 1·26 at ages 45–54) but no appreciable excess at other ages as seen in Table II (index 109). Gallstones and infective conditions of the biliary passages manifest, however, a female susceptibility with sex-ratios about 1·45 at ages up to 65 falling to 1·2 or 1·1 at later ages (index 137). For cancer of those organs the female excess appeared after 45 as already noted. No reason for the vulnerability of women to gall bladder affections in particular is known unless tight clothing could be a factor.

In the Latin American cities in 1962–64 referred to in the section on cancer of the liver complex there were 381 deaths from cholelithiasis and cholcystitis and the sex-ratios at ages 35–44, 45–54, 55–64 and 65–74 were 2·00, 1·76, 1·81, 1·91 (index 185), an even greater female excess than in England and Wales.

Pancreatic diseases except cancer show no female excess, except at advanced ages for acute pancreatis, the sequence of sex-ratios for that cause in England and Wales being 0·51, 0·72, 0·94, 0·88 at ages 35–74 increasing suddenly to 1·36 at ages over 75 (index 85).

*Cancers of the intestine* (152, 153) *and of the rectum* (154).—In the Registrar General's Statistical Review for 1935 (Registrar General, 1938) the death rates in 1911–20, 1921–30 and 1931–36 were seen to produce sex-ratios for cancer of the rectum which differed remarkably from those for cancer of the rest of the intestine. In his Table LXV the female/male ratios between standardised rates at all ages in the three periods were 0·63, 0·48 and 0·52 for the rectum, indicating pronounced female immunity whereas for other intestine they were 1·13, 1·04 and 1·00 indicating a small female excess which had disappeared by 1931–36. Ashley (1969) has again drawn attention to this curious difference which still persists in mortality statistics. Some changes in rules of classification of " recto-sigmoid " have occurred but there is no reason why they should affect the rates for the two sexes differently.

Table II shows that in 1963–66 the female/male ratio for cancer of the intestines excluding rectum was 1·20 at ages up to 54, falling to 1·05 at 55–64 after which there was no female excess (index 105). For the rectum however the sequence from 35–44 onwards was 0·92, 0·78, 0·64, 0·39, 0·55 (index 60), and this male excess is similar to that found in the upper digestive tract. Thus, as Wynne Griffith (1968) has shown by analysis of stomach cancer rates in 1958–63 in 24 countries, the female/male ratios in England and Wales fell regularly from 0·62 at 35–39 to 0·38 at 55–59 and then increased to 0·71 at 80–84 (index 45). For the oesophagus in the same period the ratio was 0·85 at ages 35–44 falling to 0·47 at 60–64 and remaining about that level. For the mouth and pharynx the ratios were 1·05 at 35–44, 0·91 at 45–49, and at subsequent 5-year groups 0·77, 0·63, 0·51, 0·38, 0·31, 0·26, 0·22 (index 58).

The reason for the contrast between the intestine from duodenum to sigmoid with its slight female excess in death rates up to age 65 and the large male excess for the rest of the digestive tract is obscure. That it was present throughout northern Europe is evident from the mean sex-ratios of female to male mortality during 1954–61 set out below for cancer of the intestine excluding the rectum (Table VIII). As was the case for cancer of the biliary passages the Netherlands showed the greatest female excess at every age after 45.

TABLE VIII.—*Mean Sex-ratios of Female to Male Mortality* 1954–61 *for Cancer of Intestine, Excluding Rectum*

| | Mean of sex-ratios of component countries in 1954–61 | | | | | Index |
|---|---|---|---|---|---|---|
| | 35– | 45– | 55– | 65– | 75+ | 45–74 |
| British Isles | 1·54 | 1·28 | 1·09 | 0·91 | 1·01 | 109 |
| Scandinavian countries | 1·08 | 1·20 | 1·03 | 0·95 | 1·02 | 106 |
| Netherlands | 1·12 | 1·40 | 1·24 | 1·09 | 1·29 | 124 |
| Belgium | 1·17 | 1·17 | 1·09 | 1·01 | 1·17 | 109 |
| Mid-European countries | 1·07 | 1·01 | 0·86 | 0·83 | 1·08 | 90 |

In Britain and Belgium there was pronounced female excess up to 65 and in Scandinavia up to 55, but the countries of mid-Europe (France, Germany, Austria, Switzerland, Italy) showed no such excess in the middle age groups.

*Anaemias, purpura and diseases of the spleen* (290–293, 296, 298, 299) all show evidence of female susceptibility at all ages in 1963–66 in England and Wales with high sex-ratios of 1·65 at 35–44 and 1·45 at 45–54, followed by a moderate excess about 1·20 after 55 (index 127). This is connected no doubt with the function of child bearing and needs no special comment.

*Diseases of the veins and embolism* (460–466) show pronounced female excess of mortality at ages 35–44 and a small excess at 45–54, probably due to delayed results of child bearing. At later ages there is some excess at 75 and over but none at the intervening ages (index 100).

*Asthma* (241) in 1963–66 presented an excess in female death rates from age 15 to 65, the successive sex-ratios at age groups from 15–24 onwards being 1·20, 1·19, 1·54, 1·31, 1·10, 0·86, 0·54 (index at 45–74 109). This is in sharp contrast with bronchitis which showed a high degree of female immunity relative to males (ratios at the same ages 0·65, 0·72, 0·60, 0·28, 0·17, 0·19, 0·31, and index 21). According to the definition of asthma in the International List as used in 1963–66, any mention of bronchitis on a death certificate along with asthma causes the death to be classified to the former disease and since such association is more frequent among men a larger proportion of deaths mentioning asthma are assigned to bronchitis (500–502) for that sex. This might account for the high sex-ratios suggesting female vulnerability at ages under 65, which may be an artefact, and the inclusion of asthma in Table II is of doubtful validity. It may be noted that owing to the difficulty of disentangling the two conditions as cause of death asthma has been transferred from the " allergic " group to become a subdivision of bronchitis in the 1968 revision of the international list.

*Malignant melanoma of the skin* (190) shows female excess in death rates in 1963–66 with a sex-ratio of 1·27 at 35–44, falling to around 1·10 between 45 and 74 and to unity at 75 and over (index 109). Other skin cancer shows a male excess and in all countries this is true of total skin cancer. In a series of 111 patients treated in South Wales for melanoma, all of them Caucasians, the female excess was found to be confined to lesions on the lower limbs (Jones *et al.*, 1968).

*Diabetes mellitus* (260).—Death rates from this disease in England and Wales show significant regional differences and the sex-ratios are by no means the same in the north as in the south. In Table IX division is made into three groups of the 15 hospital regions: *North west* (comprising Manchester, Liverpool, Leeds, Newcastle and Welsh hospital regions); *Central* (Sheffield, Birmingham, East Anglia, North East Metropolitan and North West Metropolitan regions); *Southern* (South western, Oxford, Wessex, South west Metropolitan and South east Metropolitan regions). The mean annual death rates in 1963–66 are given for each group and for comparison with the northern group the rates in Scotland for 1954–61 are also shown.

The comparisons in Table IX reveal some curious facts about the distribution of mortality from diabetes by sex and age within Great Britain. (1) Death rates of men in England and Wales increased between 1954–61 and 1963–66 by about one third at ages 35–64 and one quarter at ages over 65, whereas those of women remained steady at 35–64 and increased slightly after 65. (2) Death rates of men in the north west of England and Wales in 1963–66 were about 10 per cent higher

TABLE IX.—*Diabetes Mortality by Sex and Age in 3 Areas of England and Wales (1963–66) and in Scotland, England and Wales (1954–61)*

| | Males | | | | | Females | | | | |
|---|---|---|---|---|---|---|---|---|---|---|
| | 35– | 45– | 55– | 65– | 75+ | 35– | 45– | 55– | 65– | 75+ |
| *England and Wales* 1963–6 | | | | | | | | | | |
| Mean annual rates per million: North west | 22 | 34 | 98 | 304 | 799 | 10 | 28 | 144 | 520 | 908 |
| Central | 19 | 36 | 114 | 329 | 732 | 11 | 25 | 113 | 415 | 890 |
| South | 18 | 34 | 83 | 273 | 763 | 9 | 24 | 91 | 335 | 761 |
| All regions | 20 | 35 | 99 | 304 | 742 | 10 | 26 | 117 | 426 | 853 |
| Ratio to male rate: North west | | | | | | 0·44 | 0·82 | 1·47 | 1·71 | 1·13 |
| Central | | | | | | 0·60 | 0·70 | 0·99 | 1·26 | 1·22 |
| South | | | | | | 0·49 | 0·72 | 1·10 | 1·22 | 1·10 |
| All regions | | | | | | 0·51 | 0·75 | 1·08 | 1·40 | 1·15 |
| Ratio of North-west to South: Death rates | 1·20 | 1·02 | 1·18 | 1·11 | 1·05 | 1·22 | 1·16 | 1·55 | 1·55 | 1·19 |
| Sex-ratios | | | | | | 0·90 | 1·14 | 1·34 | 1·40 | 1·13 |
| *England and Wales* 1954–61 | | | | | | | | | | |
| Rates per million | 15 | 27 | 76 | 261 | 605 | 10 | 28 | 125 | 397 | 731 |
| Ratio to male rate | | | | | | 0·67 | 1·05 | 1·65 | 1·52 | 1·21 |
| *Scotland* 1954–61 | | | | | | | | | | |
| Rate per million | 18 | 42 | 109 | 337 | 622 | 16 | 43 | 277 | 717 | 963 |
| Ratio to male rate | | | | | | 0·93 | 1·04 | 2·53 | 2·13 | 1·55 |
| Ratio to England & Wales | 1·20 | 1·56 | 1·43 | 1·29 | 1·03 | 1·60 | 1·54 | 2·22 | 1·81 | 1·32 |

than in the south, whereas among women the north west excess was 55 per cent at 55–74 and about 20 per cent at other ages. (3) Death rates of men in Scotland in 1954–61 were about 50 per cent higher than in England and Wales at ages 45–64 and about 25 per cent higher at 35–44 and 65–74, whereas among women the excess was about 50 per cent at 35–54, 100 per cent at 55–74 and 30 per cent at 75 and over. (4) After allowing for the rise in death rates noted in (1) and assuming that the Scottish rates behaved in the same way, the differences between Scotland and Southern England when expressed in terms of the latter taken as 100 for the successive age groups were 28, 47, 59, 29, 0 for men whereas the northern excess was much greater for women, namely 77, 71, 195, 123, 42. (5) There is therefore a pronounced upward gradient in mortality from south to north in Great Britain, stronger among women at every age and particularly at ages 55–74. (6) The sex-ratios show male preponderance in all parts of England and Wales at ages up to 54, then changing to a female excess which is very pronounced in the north west at 55–74 with ratios around 1·6 but only slight in the rest of England. In Scotland the female susceptibility at ages 55–74 is still more evident with ratios about 2·3.

The regional comparison is depicted in Fig. 2, and the pattern is more suggestive of differential action of extrinsic factors on the sexes than of genetic differences in female susceptibility to diabetes. Thus the explanation might lie in a larger ingestion of sugar and other carbohydrates by middle aged women compared with men in the northern parts of Britain than in the south.

In Table X sex-ratios are shown at the five age groups between diabetes death rates in 1954–61 in 26 countries arranged in ascending order of the index at 45–74. In the first two, Ceylon and Portugal, there was no appreciable female excess at any age, and in the next, Japan, it was not present after 55. In the

Fig. 2.—Diabetes death rates for each sex in three parts of England and Wales in 1963–66 with ratios between North west and South and sex ratios at each age. (For the rates and ratios see Table IX, dividing death rates by 10.)

other 23 countries the predominant picture is a sudden change from male to female excess at 55, exceptions being Australia, U.S. Whites, England and Wales, Scotland, Netherlands, Belgium, Hungary and Israel where the change occurred earlier at about 45, and Colombia, Venezuela and U.S. Non-whites, where it had already occurred at 34. This means that in 23 of the 26 countries there was a female excess at every age after 55. The sudden change from male to female preponderance was in most cases very pronounced and subsequent changes after 55 were only slight. The most interesting age period for comparing the countries was 45–54 at which age Great Britain and Australia had developed slight female excess, Hungary and Venezuela showed about 15 per cent and Colombia, U.S.A., Netherlands, Belgium and Israel already had large female excess. In Ireland, Scandinavia, Central Europe except Hungary, Canada and New Zealand however men still had the highest death rates at this age period. The ten Latin America cities studied in 1962–64 (Puffer and Wynne Griffith, 1967) did not show any female excess until age 55 except in Cali and Sao Paulo.

The reasons for these geographical differences are obscure but some peculiarity in dietary habits of women before 55 in the Low countries, U.S.A., Colombia, Venezuela and Israel, appearing later in other countries, may account for them.

TABLE X.—*Diabetes Sex-ratios Between Mean Annual Death Rates of Females in* 1954–61 *and those of Males in* 26 *Countries Ranked in Order of the Average Ratio at Ages* 45 *to* 74

| | 35– | 45– | 55– | 65– | 75+ | Index at 45–54 |
|---|---|---|---|---|---|---|
| Ceylon | 0·83 | 0·91 | 0·75 | 0·58 | 0·36 | 75 |
| Portugal | 0·67 | 0·95 | 0·91 | 1·03 | 0·91 | 96 |
| Japan | 1·30 | 1·04 | 0·94 | 0·95 | 0·76 | 98 |
| France | 0·75 | 0·83 | 1·19 | 1·28 | 1·25 | 110 |
| Ireland (Eire) | 0·86 | 0·70 | 1·31 | 1·32 | 1·20 | 111 |
| Italy | 0·83 | 0·85 | 1·36 | 1·31 | 1·26 | 117 |
| Norway | 0·59 | 0·61 | 1·37 | 1·53 | 1·49 | 117 |
| Sweden | 0·62 | 0·70 | 1·29 | 1·56 | 1·23 | 118 |
| Denmark | 0·51 | 0·68 | 1·51 | 1·52 | 1·17 | 124 |
| Northern Ireland | 1·27 | 0·88 | 1·66 | 1·28 | 1·20 | 127 |
| Austria | 0·42 | 0·97 | 1·36 | 1·57 | 1·73 | 130 |
| Australia | 0·95 | 1·04 | 1·31 | 1·56 | 1·42 | 130 |
| New Zealand | 0·38 | 0·94 | 1·34 | 1·64 | 1·03 | 131 |
| Switzerland | 1·14 | 0·94 | 1·43 | 1·57 | 1·49 | 131 |
| Germany, F.R. | 0·78 | 0·96 | 1·49 | 1·53 | 1·52 | 133 |
| U.S.A. (White) | 0·63 | 1·57 | 1·14 | 1·49 | 1·25 | 133 |
| Canada | 0·49 | 0·96 | 1·55 | 1·50 | 1·26 | 134 |
| Colombia | 1·20 | 1·49 | 1·34 | 1·31 | 1·17 | 138 |
| Hungary | 0·91 | 1·18 | 1·45 | 1·58 | 1·66 | 140 |
| England and Wales | 0·67 | 1·05 | 1·65 | 1·52 | 1·20 | 141 |
| Israel | 0·50 | 1·71 | 1·50 | 1·11 | 1·09 | 144 |
| Finland | 0·63 | 0·75 | 1·55 | 2·11 | 2·50 | 147 |
| Venezuela | 1·03 | 1·15 | 2·11 | 2·02 | 1·25 | 176 |
| Netherlands | 0·86 | 1·47 | 1·73 | 2·20 | 1·93 | 180 |
| Belgium | 0·82 | 1·88 | 1·77 | 1·89 | 1·71 | 185 |
| Scotland | 0·93 | 1·04 | 2·53 | 2·13 | 1·55 | 190 |
| U.S.A. (Non-white) | 1·42 | 2·15 | 2·21 | 1·71 | 1·49 | 202 |

*Deaths due to violence.*—There are a few accidental causes of death to which women of certain ages are more prone than men, and although the reasons for it are plain they are shown in Table XI in order to complete the record. For utility gas poisoning and falls from one level to another the excess of female

TABLE XI.—*Sex-ratios for Death Rates from Violent Causes in* 1963–66 *Showing a Female Excess, in England and Wales*

| Kind of accident | International No. | No. of deaths at 35+ | Sex-ratio female/male at ages 35– | 45– | 55– | 65– | 75– | 85+ |
|---|---|---|---|---|---|---|---|---|
| Poisoning by utility gas | E890 | 3613 | 1·56 | 0·57 | 0·69 | 1·00 | 1·08 | 1·04 |
| Fall from one level to another (not stairs) | E902 | 1768 | 0·92 | 0·16 | 0·14 | 0·46 | 0·87 | 1·09 |
| Fall on same level | E903 | 10979 | 0·45 | 0·50 | 0·78 | 1·27 | 1·49 | 1·56 |
| Fall, kind unspecified | E904 | 4789 | 0·36 | 0·49 | 1·10 | 1·48 | 1·72 | 1·61 |
| Fire or explosion of combustible material | E916 | 2301 | 0·63 | 1·16 | 1·39 | 1·49 | 1·23 | 0·88 |

liability to a fatal issue appears only in very advanced age, and for other falls it is seen after about 60. There is an excess of fire risk for women at all ages from 45 to 84.

SUMMARY

Death rates from a small proportion of the causes in the International Classification are higher among women than among men at the same ages, and the amount of female susceptibility and the ages at which it is present in England and Wales and other countries is investigated for such causes by means of the sex-ratios.

In the 24 groups of causes involved are four sites of cancer (excluding the breast and organs peculiar to females), namely the thyroid, biliary passages, intestine other than rectum and melanoma of the skin, and for the first two of these the sex-ratios are compared with those for other diseases of the same organs.

Geographical differences are pronounced for cancers of the thyroid, biliary passages and intestine, and also for diabetes. For some of the causes of death extrinsic factors or childbearing can account for the greater susceptibility of women but for most of the others it is concluded that differences in genetic control emanating from the extra X-chromosomes probably account for greater vulnerability of females to disease in certain tissues such as the thyroid glands and the gall bladder.

### REFERENCES

Ashley, D. J. B.—(1969) *Br. J. Cancer*, **23**, 26.

Clemmesen, J.—(1964) ' Statistical Studies in Malignant Neoplasms '. Copenhagen (Munksgaard).

Copeman, W. S. C.—(1964) ' Textbook of the Rheumatic Diseases '. 3rd edition. Edinburgh and London (Livingstone).

Griswold, M. H., Wilder, C. S., Cutler, S. J. and Pollack, E. S.—(1955) ' Cancer in Connecticut, 1935–51 '. Hartford (Connecticut State Department of Health).

Jones, W. M., Williams, W. J., Roberts, M. M. and Davies, K.—(1968) *Br. J. Cancer*, **22**, 437.

Lawrence, J. S.—(1961) *Ann. rheum. Dis.*, **20**, 11.

Norwegian Cancer Society—(1964) ' Cancer Registration in Norway, Incidence in 1959–61 '. Oslo.

Puffer, R. R. and Wynne Griffith, R.—(1967) ' Patterns of Urban Mortality '. Washington D.C. (Pan American Health Organisation).

Registrar General—(1938) 'Statistical Review for 1935'. Text. London (H.M. Stationery Office).

Segi, M. and Kurihara, M.—(1966) ' Cancer Mortality for Selected Sites in 24 Countries ', No. 4. Sendai, Japan (Department of Public Health, Tohoku University School of Medicine).

Segi, M., Kurihara, M. and Tsukahara, Y.—(1966) ' Mortality from Selected Causes in 30 Countries, 1950–61 '. Tokyo, Japan (Kosei Tokei Kyokai).

Steinitz, Ruth—(1967) ' Five Years' Morbidity from Neoplasms, 1960–64 '. Israel (Ministry of Health).

Stocks, P.—(1968) *Br. J. prev. soc. Med.*, **22**, 206.

Wynne Griffith, G.—(1968) *Br. J. Cancer*, **22**, 163.


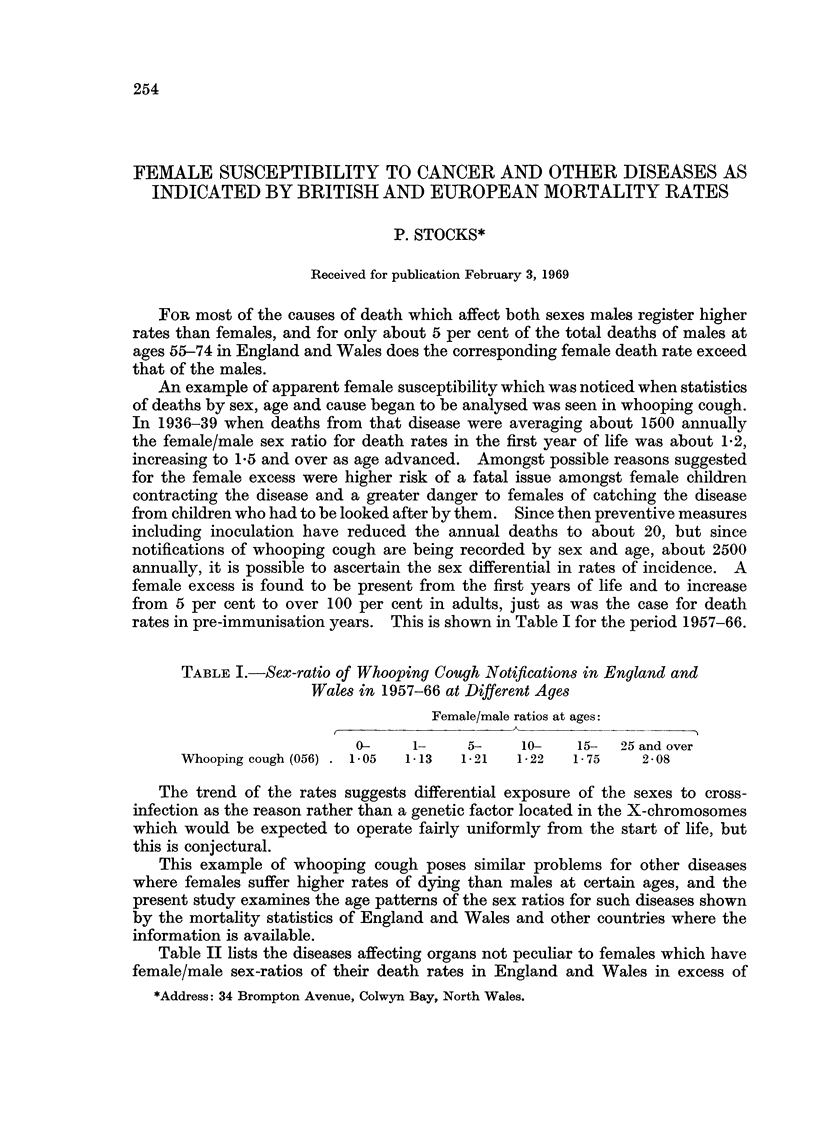

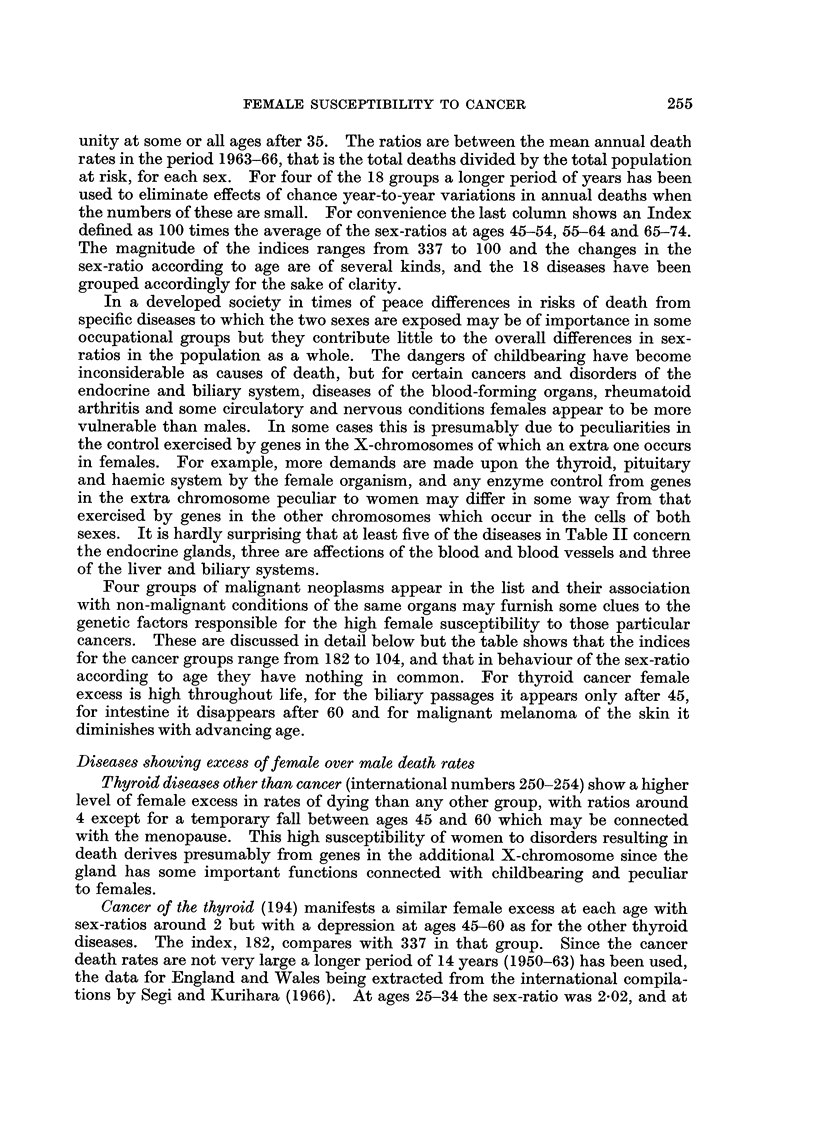

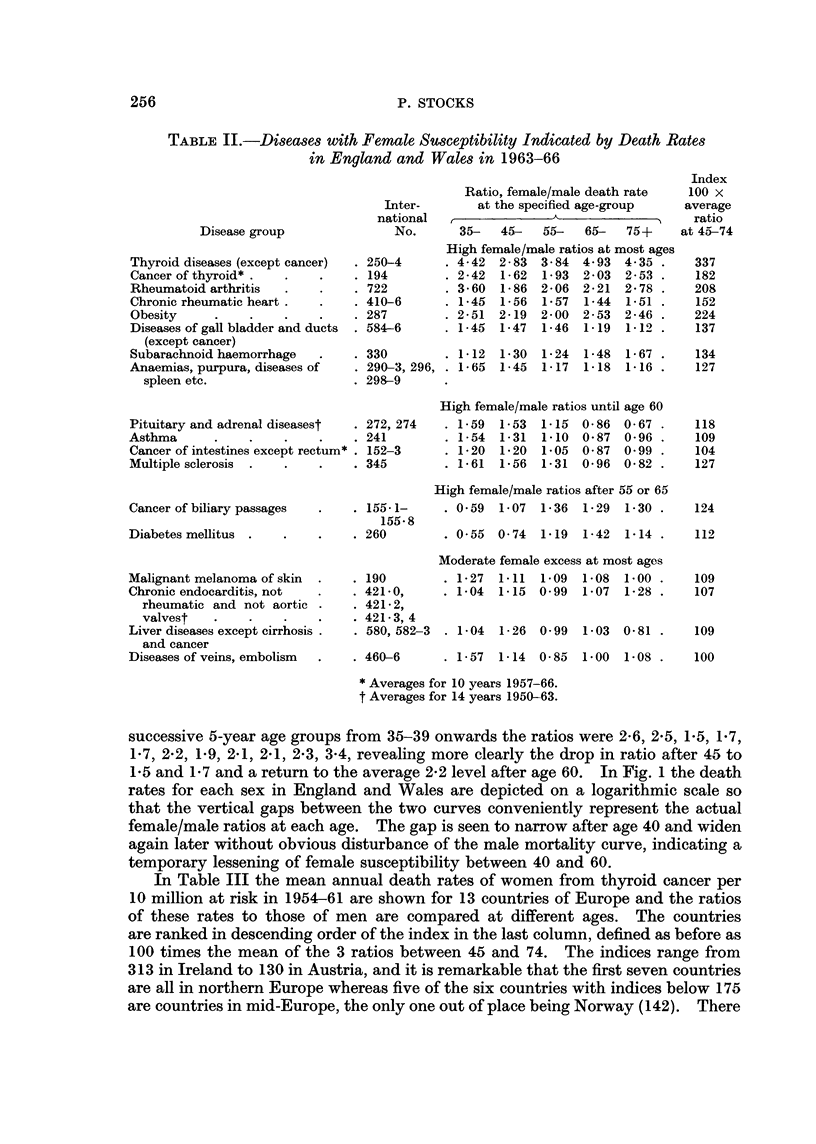

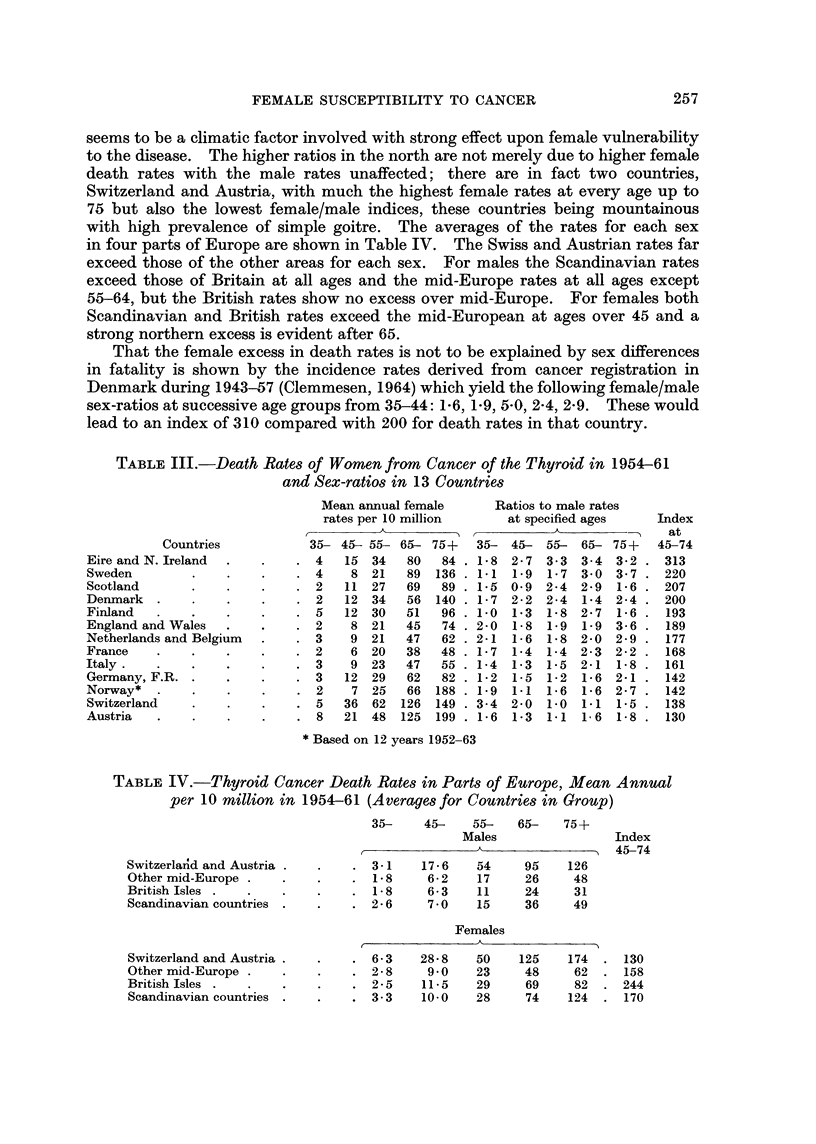

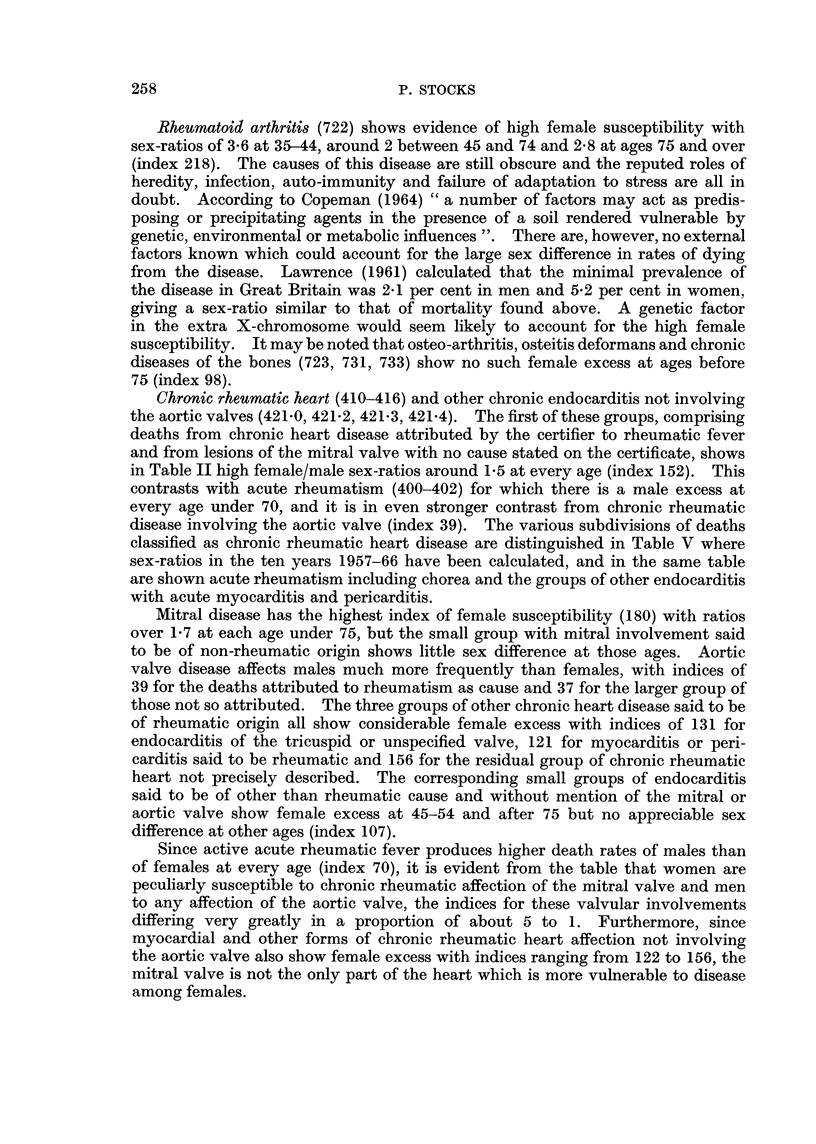

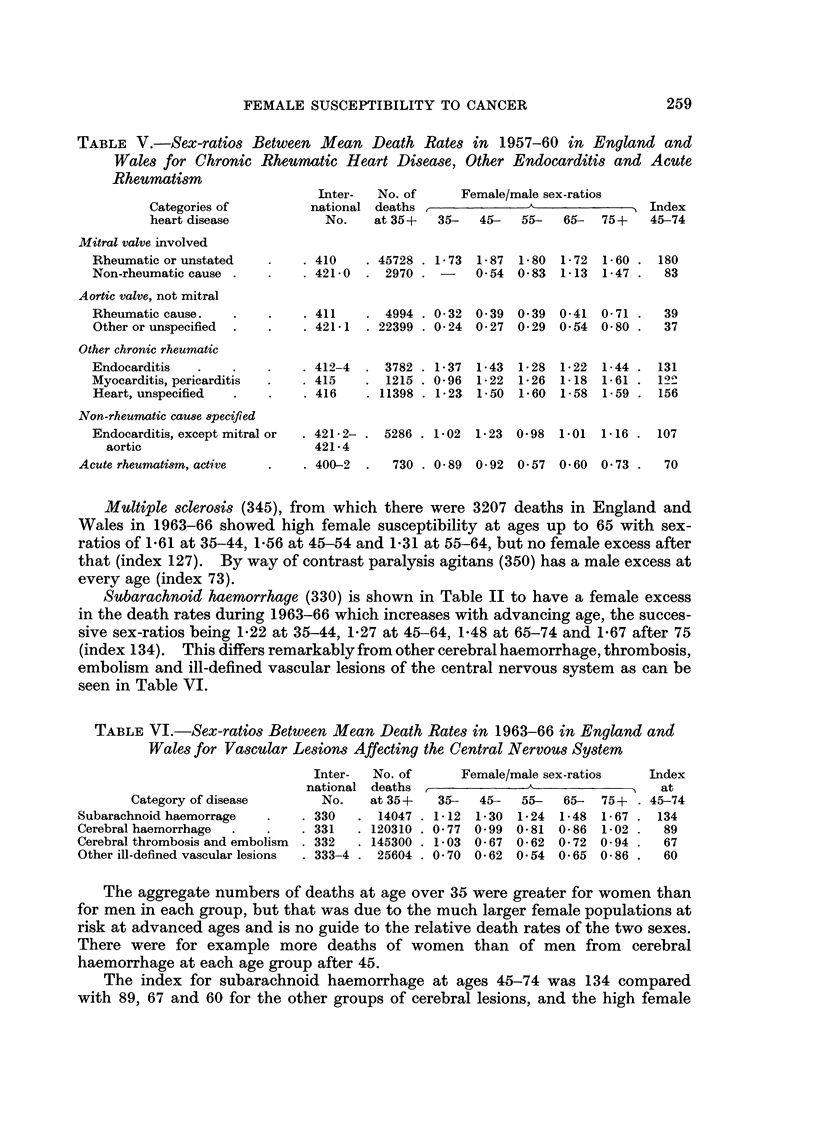

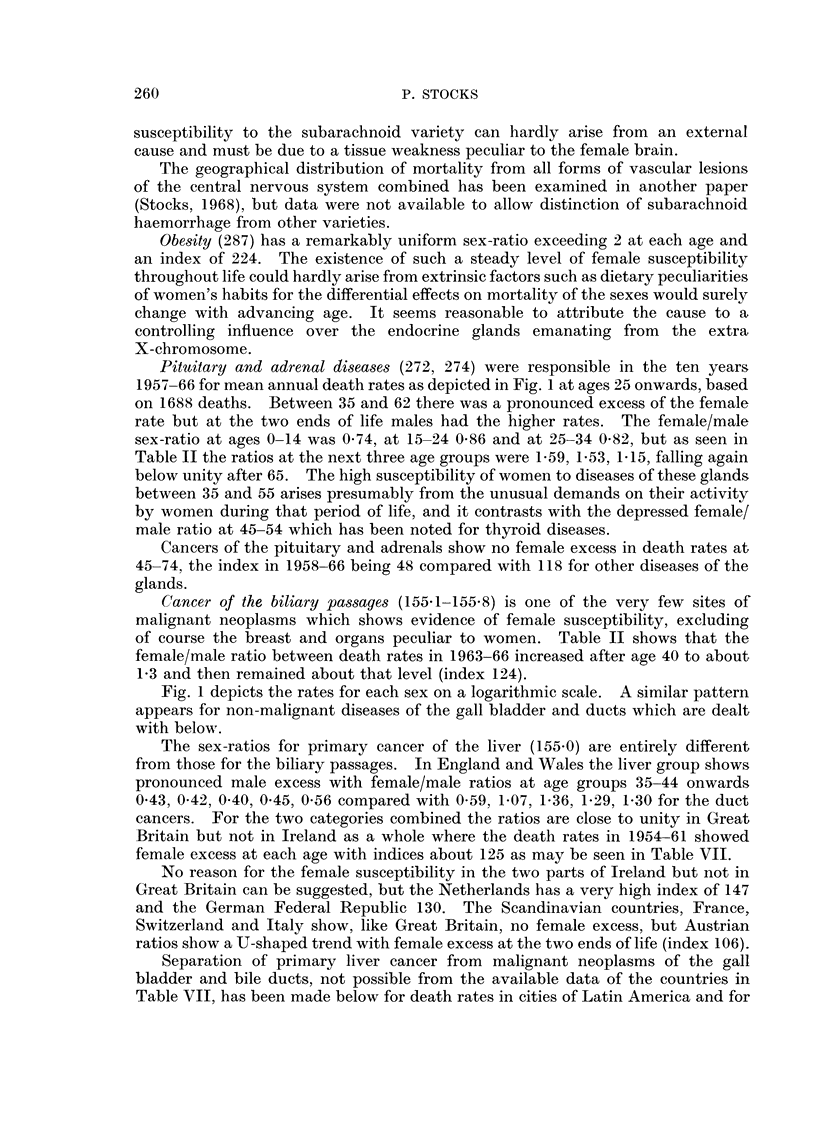

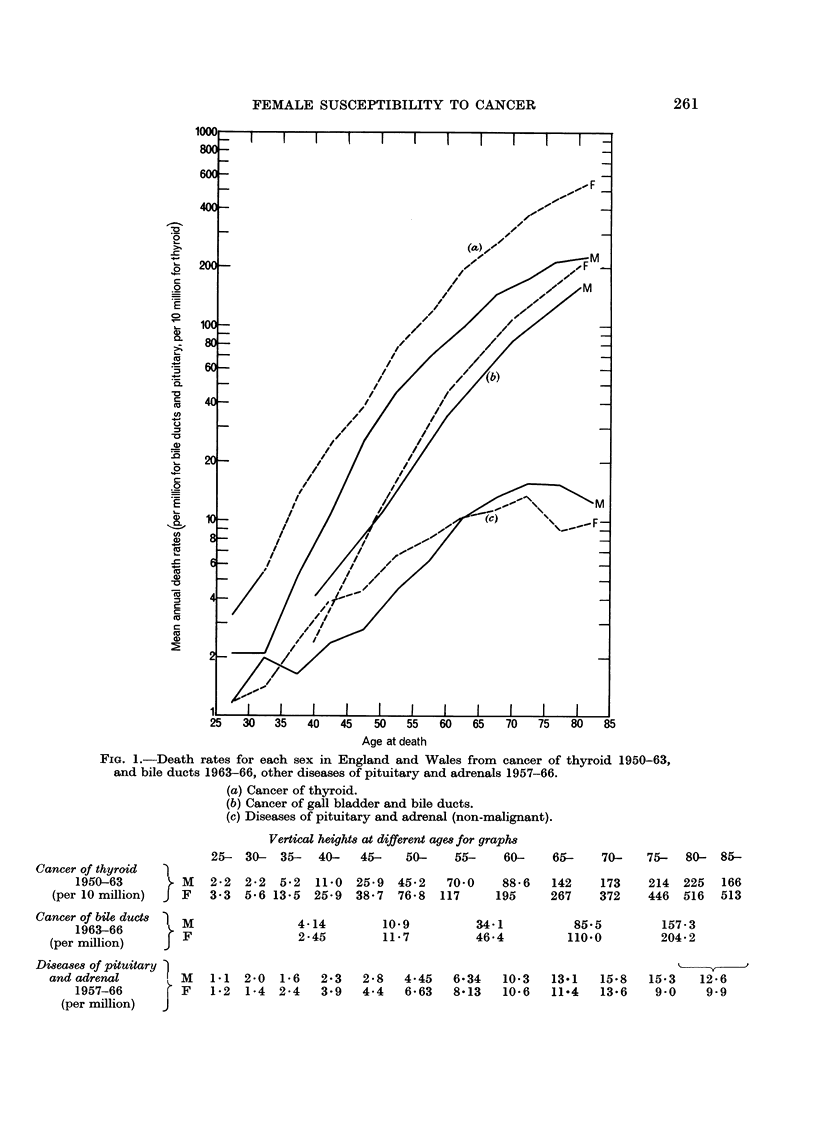

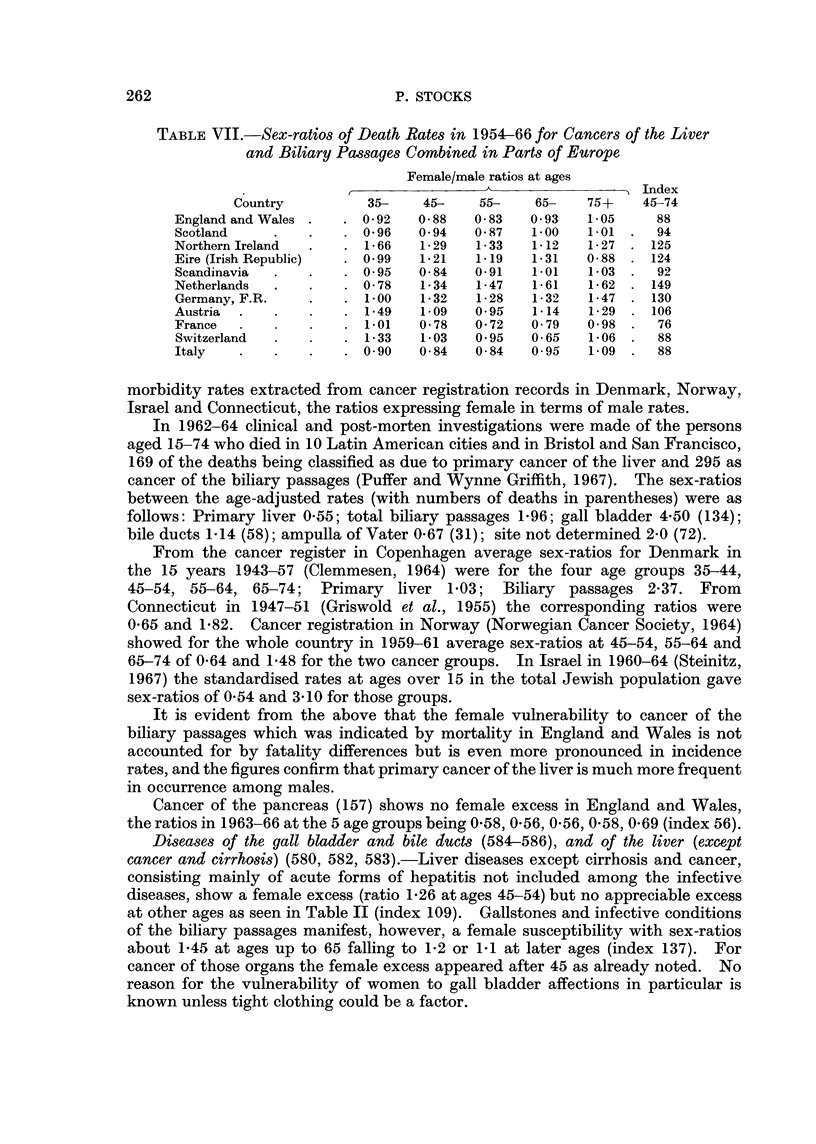

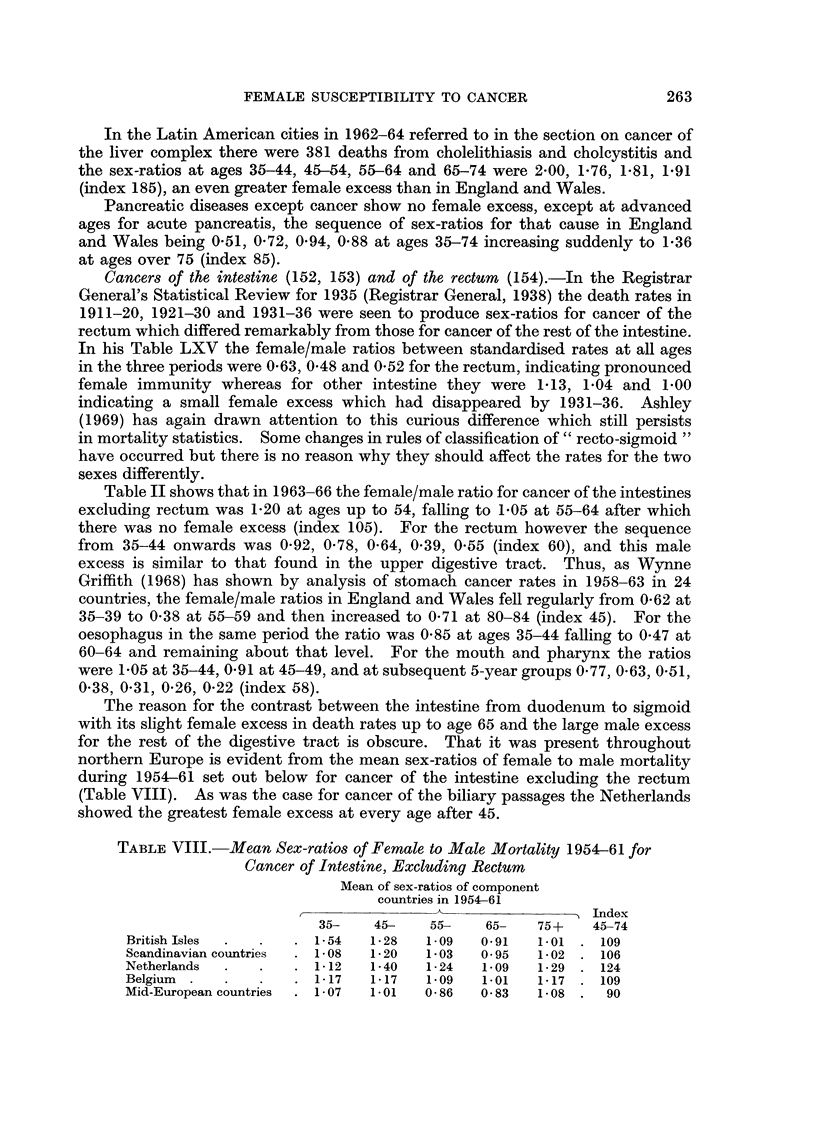

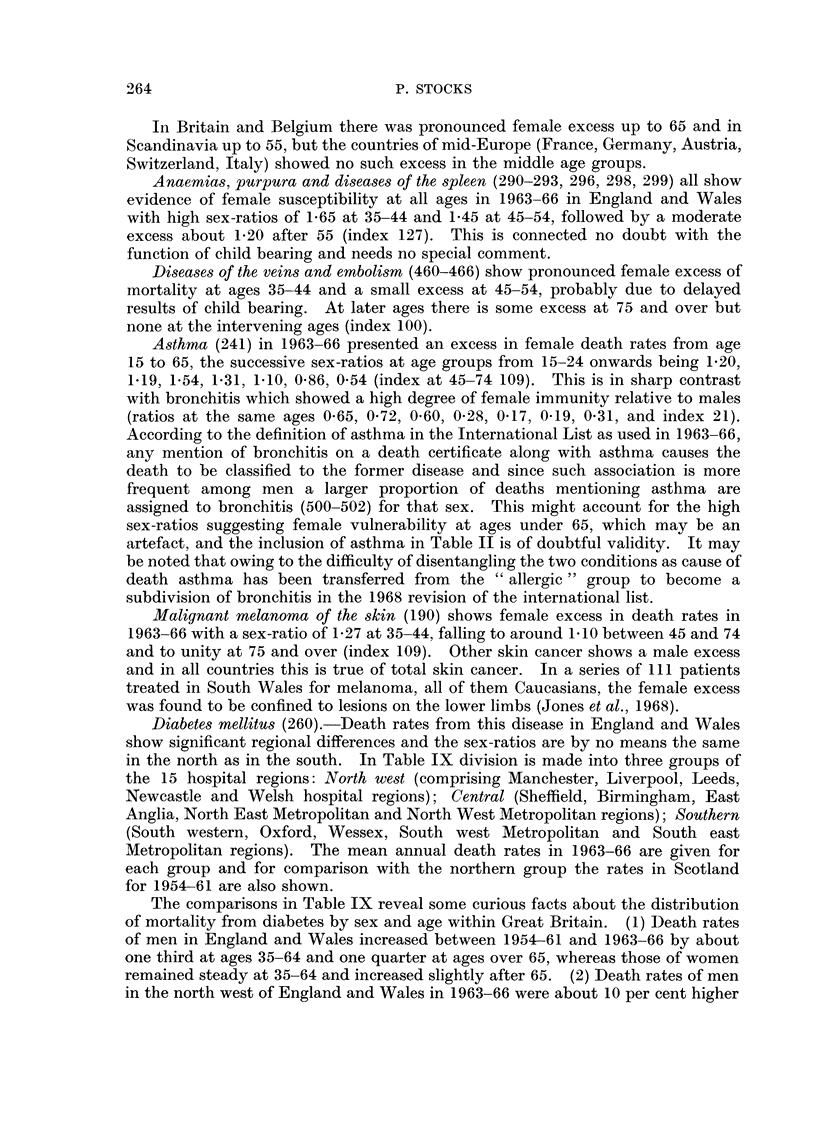

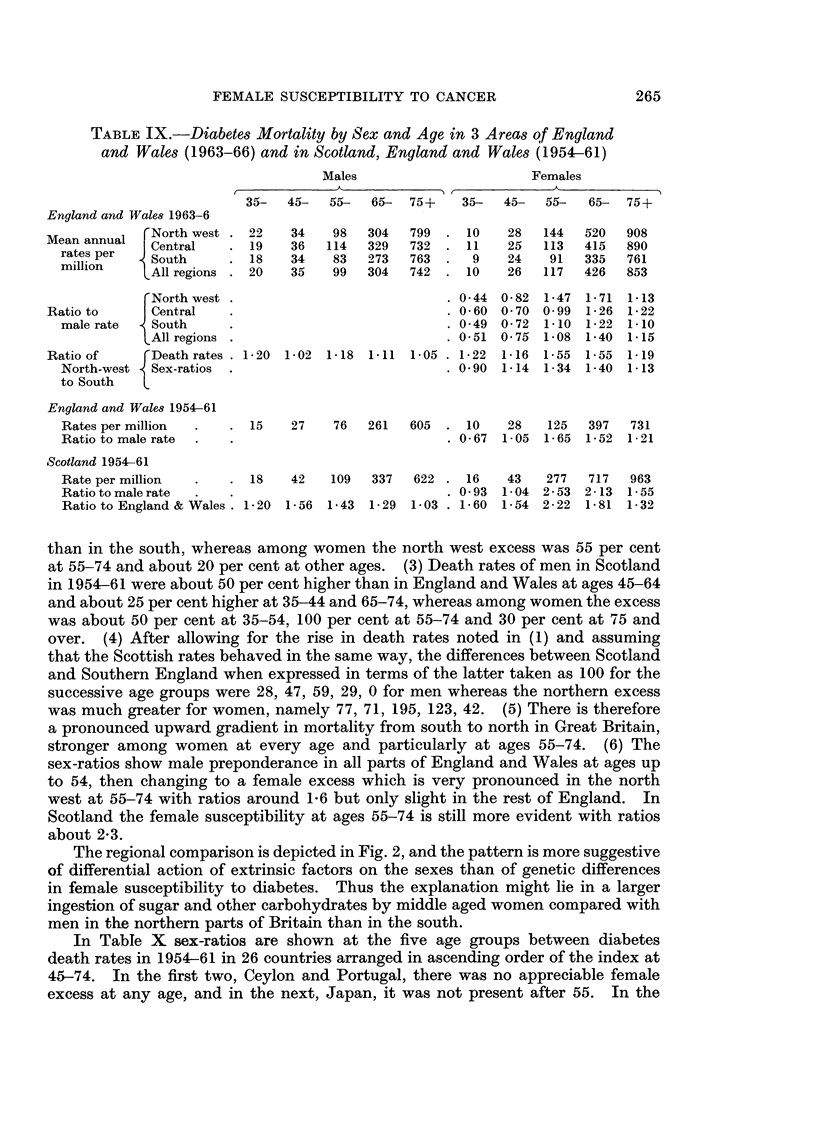

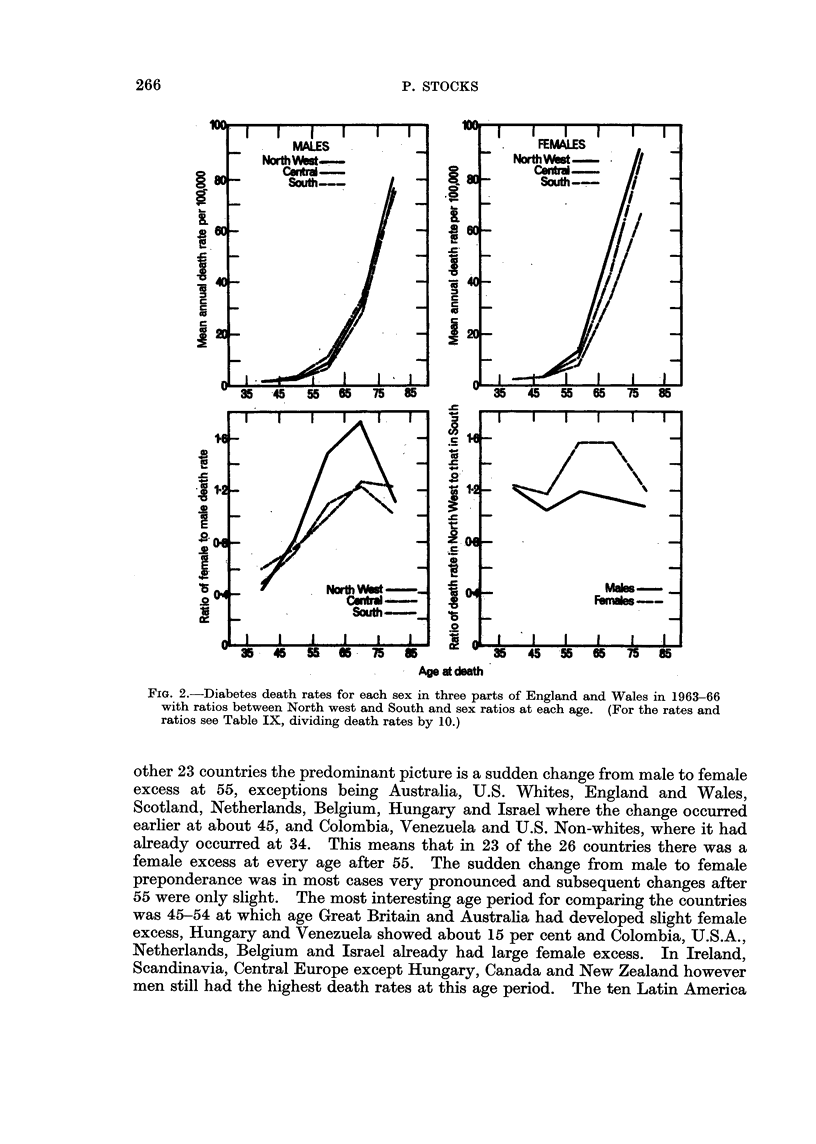

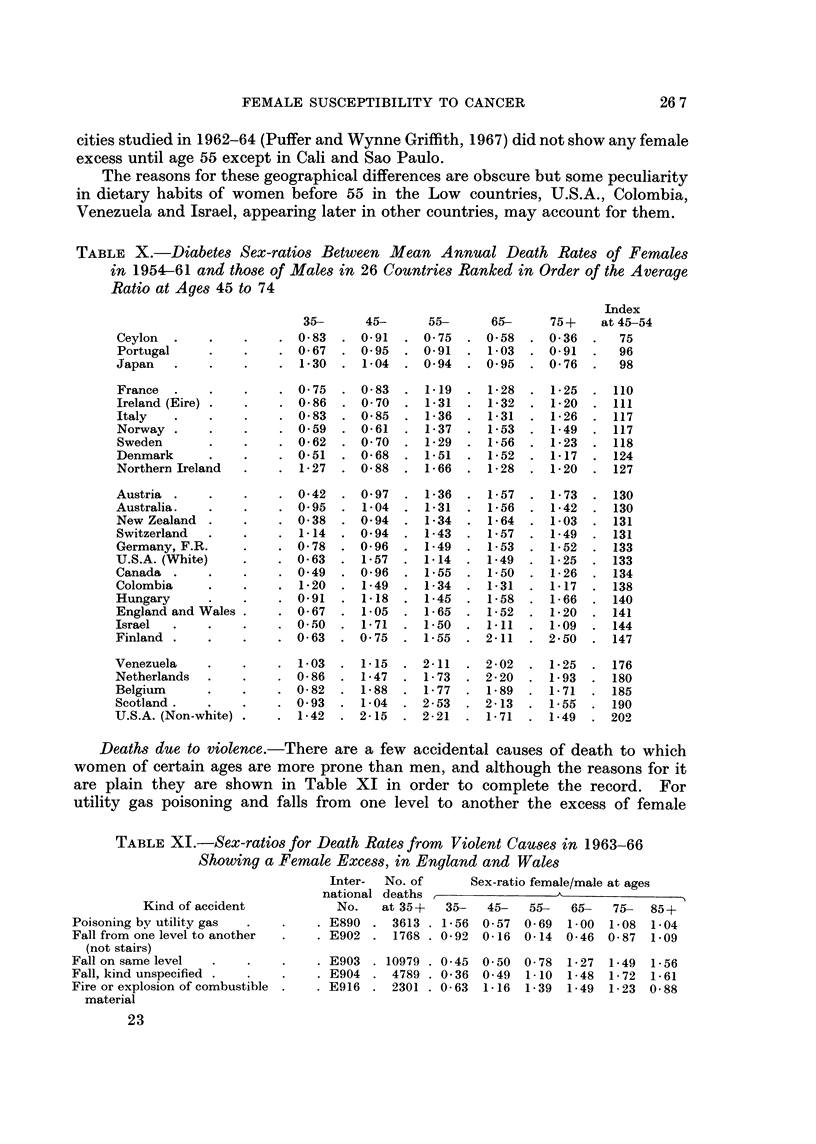

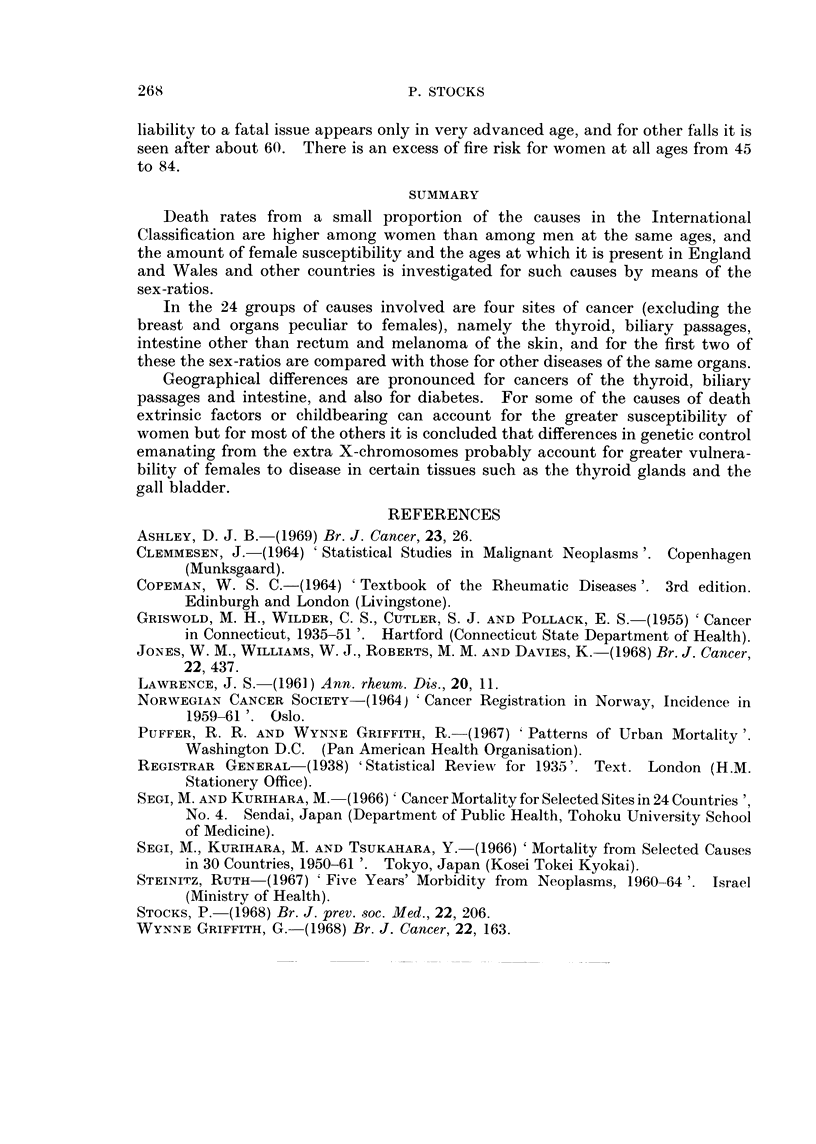


## References

[OCR_01167] Ashley D. J. (1969). Sex differences in the incidence of tumours at various sites.. Br J Cancer.

[OCR_01180] Jones W. M., Williams W. J., Roberts M. M., Davies K. (1968). Malignant melanoma of the skin: prognostic value of clinical features and the role of treatment in 111 cases.. Br J Cancer.

[OCR_01184] LAWRENCE J. S. (1961). Prevalence of rheumatoid arthritis.. Ann Rheum Dis.

[OCR_01211] Stocks P. (1968). Indications of a possible association between peptic ulcer and vascular lesions of the central nervous system.. Br J Prev Soc Med.

